# Effects of zinc supplementation on sleep quality in humans: A systematic review of randomized controlled trials

**DOI:** 10.1002/hsr2.70019

**Published:** 2024-10-06

**Authors:** Mostafa Shahraki Jazinaki, Alireza Gheflati, Mohammad Reza Shadmand Foumani Moghadam, Saeid Hadi, Maryam Razavidarmian, Masoud Yaghob Nezhad, Hale Akhtari, Mona Nematizadeh, Mohammad Safarian

**Affiliations:** ^1^ Department of Nutrition Faculty of Medicine Mashhad University of Medical Sciences Mashhad Iran; ^2^ Department of Nutrition Sciences Varastegan Institute for Medical Sciences Mashhad Iran; ^3^ Department of Health and Nutrition School of Medicine AJA University of Medical Sciences Tehran Iran

**Keywords:** sleep quality, systematic reviews as topic, zinc

## Abstract

**Background and Aims:**

Alternative therapies, such as zinc supplementation, have been explored as potential interventions for sleep disorders. However, the efficacy of zinc supplementation in improving sleep quality remains uncertain. This systematic review aims to examine the impacts of zinc supplementation on sleep quality in humans.

**Methods:**

The Web of Science, Medline, Scopus, and Google Scholar databases were comprehensively searched to find studies investigating the effect of zinc supplementation on sleep quality. After identifying relevant studies by screening, relevant data were extracted from them. The quality assessment was conducted using the Cochrane quality assessment tool.

**Results:**

This systematic review included eight studies. The interventions ranged from 4 to 48 weeks, with a daily dose of zinc supplementation varying between 10 and 73.3 mg. The majority of the evidence examined in this review pointed to the significant improvement effect of zinc supplementation on sleep quality in adults compared to the control groups. Furthermore, zinc supplementation did not have a significant effect on sleep disorders. However, there was no consensus about these findings. Also, the effect of supplementation on sleep duration in nonadults was contradictory.

**Conclusions:**

This systematic review suggests that zinc supplementation may lead to improvements in sleep quality. However, more research, primarily clinical trials, is needed to clarify the beneficial effects of zinc supplementation on sleep quality with consideration of dietary zinc intake and the Recommended Dietary Allowances of zinc (RDA) in the different populations. It is also recommended to investigate the effect of zinc supplementation on sleep quality in people with zinc deficiency in future studies.

## 1 INTRODUCTION

Sleep is a crucial physiological behavior that regulates the functioning of the human body across all age groups. Enhancing sleep quality has been linked to improvements in memory and mental health.^1^, ^2^, ^3^ However, over time, the quality of sleep has progressively declined, and diagnoses of sleep disorders such as obstructive sleep apnea (OSA), restless legs syndrome (RLS), sleep deprivation, and insomnia significantly increased.^4^ Research indicates that 64% of young adults have sleep disorders.^5^ Inadequate sleep hampers individual performance and productivity and poses a substantial financial burden on society. The estimated cost of insufficient sleep is between $280 and $411 billion in the United States and approximately $66 billion in Australia.^6^, ^7^ Furthermore, inadequate sleep is a risk factor for obesity, cardiovascular diseases, metabolic disorders, neurogenic conditions, and increased mortality rates.^8^, ^9^ The quality and quantity of sleep can be objectively evaluated either in specialized laboratories or through polysomnography tools at home.^10^ Additionally, subjective assessments can be conducted using validated measures such as the Pittsburgh Sleep Quality Index (PSQI),^11^ OSA questionnaire,^12^ and the Epworth Sleepiness Scale (ESS).^13^ The PSQI, introduced and validated by Buysse et al. in 1988, is a subjective tool for assessing sleep quality.^11^, ^14^ It consists of seven components: sleep quality, sleep latency, sleep duration, habitual sleep efficiency, sleep disturbance, use of sleeping medication, and daytime dysfunction. Each component can be assigned a score ranging from 0 to 3, with a final score range of 0−21.^11^ Contemporary medicine for sleep disorders has witnessed a growing inclination towards alternative therapies, such as meditation, herbal medicines, and dietary supplements. This might be due to the side effects associated with conventional drug treatments like benzodiazepines and melatonin agonists.^15^ Zinc supplementation has been studied in previous research and has demonstrated the potential to improve sleep in some instances.^16^, ^17^, ^18^ However, conflicting findings have been reported in other studies.^19^, ^20^, ^21^, ^22^, ^23^

Given the inconsistent results from clinical trials investigating the efficacy of zinc supplementation in improving sleep quality and the lack of a comprehensive review on this topic, this systematic review was conducted to summarize the evidence from randomized control trials related to zinc supplementation and sleep quality.

## 2 MATERIALS AND METHODS

This systematic review has been registered in the PROSPERO database with the registration code CRD42023440955.

All the steps of this review were conducted following the guidelines outlined in the Preferred Reporting Items for Systematic Reviews and Meta‐Analyses (PRISMA).^24^

### 2.1 Search strategy

A search strategy combining medical subject headings (MESH) and non‐MESH keywords was used to search Medline, Web of Science, Scopus, and, Google Scholar databases until September 2023 without any language or publication date restrictions. The search terms used were as follows:

(“zinc supplementation,” “Zn,” “Zinc”) AND (“sleep” OR “sleep quality” OR “sleep disorder” OR “insomnia” OR “sleep Apnea” OR “narcolepsy” OR “restless leg syndrome” OR “RLS” OR “parasomnias” OR “REM sleep behavior disorder” OR “Non‐24‐Hour sleep wake disorder” OR “excessive sleepiness” OR “shift work disorder” OR “periodic limb movement disorder”) AND (intervention OR “intervention study” OR “intervention studies” OR intervention* OR “controlled trial” OR “randomized” OR “randomized” OR “random” OR “randomly” OR “placebo” OR “clinical trial” OR “trial” OR “randomized controlled trial” OR “randomized clinical trial” OR “RCT” OR “double blind”).

### 2.2 Study selection

Two independent researchers (M. N) and (M. R. D) screened the articles obtained from the initial search based on their titles and abstracts. Eligibility criteria included: (a) interventional studies; (b) human studies; (c) investigation of zinc supplementation as a single or combination therapy; (d) examination of sleep quality and sleep disorders as main outcomes. This review was not restricted to a particular gender or age category.

Exclusion criteria included: (a) observational studies, review articles, letters to the editor, and short communications; (b) unavailability of the full text of the article; (c) animal studies; (d) studies did not have an appropriate control group.

### 2.3 Data extraction

The relevant data from the included studies were independently extracted by two researchers, (A. Gh.) and (M. Sh. J) The extracted data included the following: First author, publication year, country of study, number and gender distribution in each group, study design, mean age of each group at baseline, duration of intervention, details of the intervention for each group, zinc dosage, participant characteristics, main outcomes reported in the eligible trials, and main results of each study.

### 2.4 Risk of bias and quality assessment

The risk of bias and quality of the included studies was assessed using the Cochrane Interventional Article Quality Assessment Tool.^25^ Two researchers, (S. H.) and (M. Sh. J.) conducted the quality assessment independently. The risk of bias was evaluated in seven domains: random sequence generation, allocation concealment, selective reporting, blinding of participants and outcome assessment, incomplete outcome data and other potential sources of bias. The assessment of possible bias depends on the score obtained through the mentioned domains, which is divided into yes (low risk of bias), no (high risk of bias), and unclear (uncertain risk of bias). Article quality was classified as poor, fair, or good if the <2, 2, and ≥3 domains were rated low‐risk, respectively. Disagreements were resolved by consulting the third author until a consensus was reached (M. S.).

## 3 RESULTS

### 3.1 Study selection

**Figure 1:**
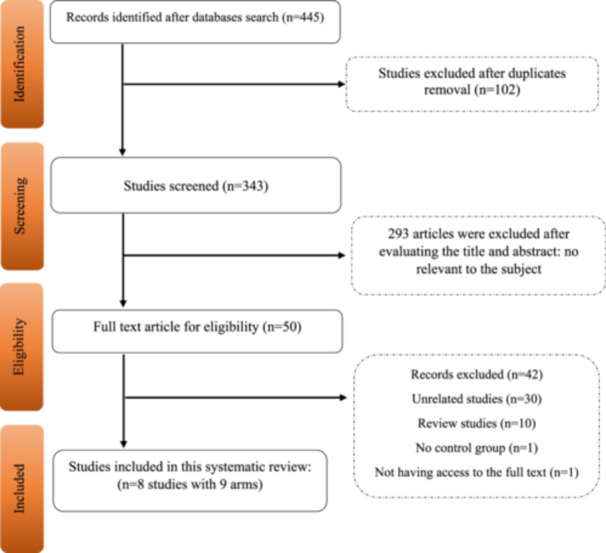
Flowchart of study selection for inclusion trials in the systematic review.

A total of 445 articles were initially identified in the systematic review. After removing duplicates, 343 articles were screened using their titles and abstracts. As a result, 293 articles were excluded as they were unrelated to the purpose of this review. The full text of the remaining 50 articles was reviewed. Finally, eight studies (with nine arms) were eligible for inclusion in this systematic review. Figure 1 illustrates the study selection process according to the PRISMA diagram.

### 3.2 Study characteristics

This review included data extracted from eight studies.^16^, ^17^, ^18^, ^19^, ^20^, ^21^, ^22^, ^23^ The included studies were published between 2009,^19^ and 2022.^17^, ^18^ Among the included studies, five were conducted in Iran,^16^, ^17^, ^18^, ^20^, ^22^ one in Spain,^23^ one in South Korea,^21^ and one in the United States.^19^ Five studies used single therapy interventions,^16^, ^17^, ^18^, ^19^, ^22^ while three^20^, ^21^, ^23^ used combination therapy.^20^, ^21^, ^23^ The interventions were conducted on different populations, including older adults,^18^ adults,^16^, ^17^, ^20^, ^22^, ^23^ children^21^ and infants.^19^

The intervention duration ranged from 4,^16^ to 48 weeks,^19^ with a daily dose of zinc supplementation varying between 10,^19^, ^23^ and 73.3 mg/day (220 mg zinc sulfate every 72 h).^16^ Table 1 provides more detailed information on the characteristics of the included studies.

**Table 1:** Characteristics of randomized controlled clinical trials included in the present systematic review.

First author Publication year Country	Number and gender distribution (f/m) in each group	Study design	Mean age of each group at the baseline (years)	Baseline serum zinc levels (mg/L)	Dietary zinc intake (mg/day)	Duration of intervention (weeks)	Dose	Intervention group	Comparison group	Notes about participant	Outcomes	Main outcome results
Afzali et al.^18^ Iran	T:150 **IN:75** M:29 F:46 **C:75** M:18 F:57	Parallel randomized clinical trial	IN: 66.8 ± 7.1 C: 66.1 ± 5.8	IN: 65.1 ± 17.1 C: 68.6 ± 10.1	NR	10 weeks	30 mg/day as zinc	Zinc supplementation	No receive any supplementation	Older Adults	PSQI	The quality of sleep in the intervention group improved significantly in all of the PSQI domains compared to the control group. Also, there was a significant and negative correlation between sleep quality scores and serum zinc levels after the intervention.
Haddadian‐Khouzani et al.^17^ Iran	T:75 **IN:38** M:26 F:12 **C:37** M:24 F:13	Parallel double‐blind randomized clinical trial	IN: 49.23 ± 15.35 C: 51.21 + 13.76	IN: 81.9 ± 16.8 C: 82.3 ± 18.2	IN: 9.2 ± 3.5 C: 8.1 ± 3.4	12 weeks	30 mg/day zinc gluconate	zinc gluconate supplementation	Placebo	Haemodialysis patients	PSQI	The quality of sleep in the group supplemented with zinc improved significantly compared to the control group.
Marrero et al.^23^ Spain	T:50 IN:24 C:26 Gender distribution was not reported	Randomized, Double‐Blind, Placebo‐Controlled Trial	IN: 51.0 ± 10.2 C: 53.7 ± 9.6	IN: 122.0 ± 6.3 C: 114.7 ± 3.9	NR	16 weeks	10 mg/day ZINC +1Mg melatonin	ZINC supplementation +melatonin	Placebo	ME/CFS patients	PSQI	There was no significant change in any of the domains of PSQI in the intervention group compared to the control group.
Jafari et al.^22^ Iran	57 IN:27 C:30	Randomized, double‐blind, placebo‐controlled trial	IN: 23.04 ± 2.97 C: 22.53 ± 1.85	IN: 75.5 ± 11.7 C: 85.5 ± 26.7	IN: 7.8 ± 1.1 C: 8.00 ± 0.9	12 weeks	30 mg/day of elemental zinc	Zinc gluconate tablets	Placebo	Young women with premenstrual syndrome aged 18–30 years old	PSQ	No significant change was detected in the sleep quality of the supplemented group compared to the control group.
Gholipour Baradari et al.^16^ Iran	T:53 **IN:27** M:3 F:24 **C:26** M:1 F:25	Double Blinded Randomized Controlled Trial	IN: 30.93 ± 5.48 C: 31.5 ± 5.46	IN: 60.1 ± 16.5 C: 59.6 ± 16.6	NR	4 weeks	220 mg zinc sulfate capsules every 72 h	Zinc sulfate capsules	Placebo	ICU Nurses	PSQI Poor Sleep Proportion	The quality of sleep in the group supplemented with zinc improved significantly compared to the control group. However, there was no significant difference in the poor sleep proportion changes between the two groups.
KIM et al.^21^ Korea	T:41 **IN:22** M:13 F:9 **C:19** M:8 F:11	randomized clinical trial	IN: 5.36 ± 3.03 C: 5.89 ± 2.03	IN: 96.3 ± 9.3 C: 93.3 ± 6.5	NR	8 weeks	12 mg/day as zinc	Zinc oxide tablets + oral antihistamines + topical moisturizers	Antihistamines + topical moisturizers	Atopic Dermatitis Children patients with low hair zinc levels	VAS score for sleep disturbance	There was no significant change in VAS scores for sleep disturbance in the zinc supplementation group compared to the control group.
Ranjbar et al.^20^ Iran	T:38 **IN:21** M:1 F:20 **C:17** M:3 F:14	Double blind randomized clinical trial	IN: 37 ± 9 C: 37.5 ± 8	IN: 100 ± 15 C: 106 ± 17	IN: 6.5 ± 2.1 C: 7.6 ± 2.2	12 weeks	25 mg zinc/day	Zinc + SSRIs	Placebo + SSRIs	Patients with Major Depression aged 18‐55 years	Sleep duration (hours per day)	During the intervention, there was no significant change in the sleep duration of the zinc supplementation group compared to the control group.
Kordas et al.^19^ USA A	T:434 **IN:219** M:103 F:116 **C:215** M:108 F:107	Randomized, placebo‐controlled trials	IN: 12.9 ± 3.9 (M) C: 12.1 ± 3.8 (M)	NR	NR	12 months	10 mg/day elemental zinc(Infants less than 1 year old received half of this dose)	Zinc supplementation	Placebo	Infants from Zanzibar	Maternal Reports of infants Sleep patterns	Zinc supplementation had no significant impact on total sleep duration compared to the control group.
Kordas et al.^19^ USA B	T:277 **IN:125** M:52 F:73 **C:152** M:70 F:82	Randomized, placebo‐controlled trials	IN: 10.4 ± 4.2 (M) C: 11.2 ± 4.1 (M)	NR	NR	12 months	10 mg/day elemental zinc(Infants less than 1 year old received half of this dose)	Zinc supplementation	Placebo	Infants from Nepal	Maternal Reports of infants Sleep patterns	Zinc supplementation significantly increased the total sleep duration compared to control group.

*Note*: Bolds indicates number of participants in each of the intervention (IN) and control (C) groups.

Abbreviations: IN, intervention; C, control; M, male; F, female; T, total; IN, intervention group; C, control groups; M, males; F, females; USA, united states of America; U.K, United Kingdom, NR, not reported; VAS, Visual analog scales; ME/CFS, Myalgic Encephalomyelitis/Chronic Fatigue Syndrome; M, months.

### 3.3 Effect of zinc supplementation on sleep quality

Afzali et al.^18^ Haddadian‐Khouzani et al.^17^ Castro‐Marrero et al.^23^ Jafari et al.^22^ Gholipour Baradari et al.^16^ investigated the effect of zinc supplementation on sleep quality using PSQI in adults. In a study by Afzali et al. daily supplementation with 30 mg of zinc in older adults for 10 weeks significantly improved sleep quality compared to the control group.^18^ Similarly, Haddadian‐Khouzani et al. found that supplementation with 30 mg of zinc gluconate in dialysis patients for 12 weeks significantly improved sleep quality compared to the placebo.^17^ However, Castro‐Marrero et al. conducted a study on Myalgic Encephalomyelitis/Chronic Fatigue Syndrome (ME/CFS) patients and found that combination therapy of 10 mg of melatonin with 10 mg of zinc over 16 weeks did not lead to significant changes in any of the domains of the PSQI.^23^

In a study by Jafari et al. on young women with premenstrual syndrome, supplementation with 30 mg of zinc daily for 12 weeks did not significantly change sleep quality compared to the control group.^22^ On the other hand, Gholipour Baradari et al. conducted a study on ICU nurses and found that supplementation with 220 mg of zinc sulfate every 72 h significantly improved subjective sleep quality, and total sleep quality score measured by the PSQI compared to placebo.^16^

### 3.4 Effect of zinc supplementation on sleep duration

#### 3.4.1 Effect of zinc supplementation on sleep duration in adults

When it comes to the effect of zinc supplementation on sleep duration, findings have been mixed in different age groups. Gholipour Baradari et al. found that supplementation with 220 mg of zinc sulfate every 72 h did not result in a significant change in sleep duration compared to the control group.^16^ Similarly, Ranjbar et al. conducted a study on patients with major depression and found that receiving 25 mg of zinc supplement daily for 12 weeks did not lead to a significant change in sleep duration compared to the control group.^20^

#### 3.4.2 Effect of zinc supplementation on sleep duration in non‐adults

Kordas et al. conducted a study in two phases on infants from Zanzibar and Nepal. This trial showed that zinc supplementation significantly increased the total sleep duration in Nepalian infants compared to placebo while having no significant impact on total sleep duration in Zanzibarian infants.^19^

### 3.5 Effect of zinc supplementation on sleep disorders

The effect of zinc supplementation on sleep disorders has also been examined. Kim et al. conducted a study on atopic dermatitis children patients with low hair zinc levels and found that combination therapy of zinc, antihistamines, and topical moisturizers did not significantly improve the sleep disturbance score compared to the control group.^21^

### 3.6 Risk of bias and quality assessment

The quality assessment of the included articles was conducted using the Cochrane quality assessment tool.^25^ Seven studies were good,^16^, ^17^, ^18^, ^19^, ^21^, ^22^, ^23^ and one was of fair quality.^20^ Table 2 displays the assessment of the risk of bias in each domain of the Cochrane quality assessment tool.

**Table 2:** Study quality and risk of bias assessment using Cochrane Collaboration's tool: (L) low risk of bias, (U) unclear risk of bias, (H) high risk of bias.

First author	Random sequence generation	Allocation concealment	Blinding (participants and personnel)	Blinding (outcome assessment)	Incomplete outcome data	Selective reporting	Other sources of bias	General quality
Haddadian‐Khouzani et al.^17^	L	L	L	U	L	L	U	Good
Afzali et al.^18^	L	U	H	L	L	L	U	Good
Marrero et al.^23^	U	L	L	U	L	L	U	Good
Jafari et al.^22^	U	L	L	U	L	L	U	Good
Gholipour Baradari et al.^16^	L	L	L	U	L	L	U	Good
Kim et al.^21^	U	L	H	U	L	L	U	Good
Ranjbar et al.^20^	U	U	L	U	U	L	U	Fair
Kordas et al.^19^	L	L	U	U	L	L	U	Good

## 4 DISCUSSION

Recommended Dietary Allowances (RDAs) for Zinc in both genders from birth to 6 months is 2 mg/day, between 7 months and 3 years old is 3 mg/day, between 4 and 8 years is 5 mg/day, and between 9 and 13 years is 8 mg/day. Furthermore, in males aged 14 years and older zinc RDA is 11 mg/day, and in females from 14 to 18 years old, it is 9 mg/day, and after that, it is 8 mg/day.^26^ The majority of studies that have examined the impact of zinc supplementation at doses ≥ 30 mg/day (more than RDA) in adults have reported a significant improvement in sleep quality.^16^, ^17^, ^18^ From studies with 30 mg/day zinc intervention, only Jafari et al. did not report a significant change in the sleep quality of young women with premenstrual syndrome.^22^ Among the included studies, there is limited evidence to support the impact of zinc on the duration of sleep, except for infants aged 5−18 months.^19^ It is worth noting that only one study has investigated the combined therapy of zinc supplementation with sleep disorders, and no significant impact was found. This highlights the need for further investigation in this area. In the study by Afzali et al. daily supplementation with 30 mg of zinc for 10 weeks significantly improved the sleep quality (based on PSQI) of older adults.^18^ Also, the same dosage and 12 weeks of intervention in dialysis patients also showed a significant improvement in sleep quality (based on PSQI).^17^ In another study on ICU nurses, supplementation with 220 mg/72 h (about 73 mg/day) of zinc sulfate significantly improved subjective sleep quality, and total sleep quality score (sleep quality was measured by PSQI).^16^ However, the interventions performed with 30 mg/day zinc on young women with premenstrual syndrome,^22^ 12 mg/day zinc on atopic dermatitis children patients with low hair zinc levels,^21^ and 25 mg/day zinc on patients with major depression did not lead to a significant effect on sleep quality.^20^

A study conducted by Ewing et al. showed that receiving 185.4 mg/day of zinc sulfate in children patients with atopic eczema did not lead to significant improvement in sleep disturbance.^24^ A case‐by‐case review of each study highlights the importance of study design parameters such as sample size, intervention dosage, dietary intake of zinc, tools used to measure sleep, population characteristics including health status and age which can impact the results of trials. Furthermore, some studies with similar intervention characteristics have reported a significant impact of zinc supplementation on at least one aspect of sleep.^17^, ^18^, ^19^ Although the consumption of oral zinc is usually considered nontoxic, its excessive consumption can lead to symptoms related to toxicity such as fatigue, lethargy, vomiting, and epigastric pain.^27^ Therefore, it is recommended to take zinc supplements in an appropriate therapeutic dose and with caution.

The studies that were conducted with the aim of evaluating the effect of zinc supplementation on sleep quality in children and infants did not have a significant effect on most factors related to sleep, except for the increase in sleep duration,^19^ while zinc supplementation in adults often led to an improvement in sleep quality. Among the included studies, two studies intervened with zinc gluconate, with a similar dose, one led to an improvement in sleep quality,^17^ while the other did not.^22^ This may be because the study conducted by Jafari et al. was conducted only on women, while another study conducted by Haddadian‐Khouzani et al. was conducted on both sexes. Although further interpretation of the obtained results was not possible due to the limitation in the number of included studies. However, Mariangela Rondanelli et al. in 2011 examined the effects of zinc, melatonin, and magnesium on primary insomnia in residents of a long‐term care center in Italy and reported an improvement in sleep quality that was measured by using the PSQI, and the Leeds Sleep Evaluation Questionnaire among those receiving the supplement (contains 5 mg melatonin, 225 mg magnesium, and 11.25 mg zinc).^28^ Furthermore, Samad et al. reported obese patients with sleep deficits had significantly lower levels of serum zinc compared to nonobese individuals with normal sleep.^29^ Furthermore, in another observentional study reported a significant and direct correlation between serum zinc levels and sleep duration in hours (self‐administered questionnaires that assessed the sleep duration) among ageing men.^30^

One cohort study investigated the relationship between the quality of sleep and serum zinc concentrations in children.^31^ In the first phase of this study, which was conducted when participants aged 3–5 years old, no significant relationship was detected between serum zinc concentration and sleep quality as measured with sleep items in the Chinese version of the Child Behavior Check List, while in their pre‐adolescent age, there was a significant association between serum zinc levels and sleep quality as measured with the Chinese version of the PSQI (OR = 0.559, *p* = 0.049, respectively). Furthermore, the study's longitudinal analysis revealed that low zinc concentrations predicted reduced sleep quality (OR = 0.358, *p* = 0.020) and poor sleep efficiency (OR = 0.186, *p* = 0.000) in these children at their preschool age.^31^ These findings were further supported by clinical trials.^16^, ^17^, ^18^ The study conducted by Zhang et al. to evaluate the relationship between zinc concentration and sleep quality in healthy Jinan residents in China has shown that those who slept for 7−9 h a night, considered to be “normal,” had the highest concentration of serum zinc.^22^ Although a number of past studies have reported a significant relationship between serum zinc levels and sleep quality, as well as the effect of zinc supplementation on improving sleep quality, no study has been conducted to evaluate the effect of zinc supplementation on sleep quality in people with zinc deficiency.

Another piece of evidence comes from studies on zinc‐rich food that contain 15 mg of zinc per day (more than the RDA for dietary zinc intake). These studies found that such diets not only reduced the time it took to fall asleep but also improved sleep efficiency.^32^ Moreover, the consumption of zinc‐enriched yeast foods and astaxanthin oil significantly reduced sleep onset latency.^32^

Zinc is absorbed throughout the small intestine and had a concentration‐dependent absorption. The highest rate of zinc absorption occurs in the jejunum; zinc is transported through a carrier‐mediated mechanism. The presence of phytate, a substance found in grains, corn, and rice, significantly reduces the absorption of zinc from composite meals.^33^ The phytate forms that have these adverse effects are inositol hexaphosphates and pentaphosphates. However, low phosphate levels have negligible or no influence on zinc absorption.^34^

One of the possible mechanisms can be through the regulation of neurotransmitters.^27^, ^35^ Zinc is known to modulate the activity of neurotransmitters such as glutamate and gamma‐aminobutyric acid (GABA), which play key roles in sleeping. Zinc acts as a cofactor in the synthesis and metabolism of these neurotransmitters.^27^ Studies have shown that zinc deficiency can lead to alterations in glutamate and GABA levels, which may disrupt sleep and contribute to insomnia.^27^, ^35^ Furthermore, zinc is involved in the synthesis of melatonin which plays a key role in the circadian rhythm and sleeping cycle. Studies have shown that zinc supplementation can increase melatonin levels, potentially leading to improved sleep duration and quality.^36^, ^37^ Furthermore, zinc deficiency has been associated with dysregulation of other hormones involved in sleep such as growth hormones, which may further contribute to sleep disturbances.^38^ In addition, zinc is implicated in the functioning of the immune system.^27^ Sleep and the immune system are closely interconnected.^39^ Zinc is known to promote the immune system,^27^ when the body is deficient in zinc, immune function can become compromised, which may increase its susceptibility to infections and inflammation, which may disrupt sleep patterns.^27^, ^39^ Moreover, zinc has antioxidant properties and can protect against oxidative stress.^37^ Oxidative stress has been implicated in sleep disturbances and disorders such as insomnia. By acting as an antioxidant, zinc may help to reduce oxidative damage, promoting better sleep quality.^37^, ^39^

One strength of this systematic review is that it is the first of its kind to focus on the effect of zinc supplementation on sleep quality. Furthermore, major trials that included in this review had good quality (except ranjbar et al.), enhancing the credibility of their findings. However, there are several limitations to consider. The number of included studies and participants was relatively small, which may impact the generalizability of the findings. Additionally, the diversity of the populations studied (from infants to adults) and the use of non‐identical indicators to evaluate sleep quality, duration, and disorders introduce further potential sources of variability. Also, the included studies did not consider the RDA for zinc intake in the participants' population as a factor that may influencing the results.

## 5 CONCLUSION

This review revealed that zinc supplementation may have a beneficial effect on sleep quality in adults and nonadults. Furthermore, the majority of studies that have investigated the effects of zinc supplementation at doses exceeding 30 mg/day over 12 weeks or more in adult populations reported a significant improvement in sleep quality. Although there are promising findings regarding the potential benefits of zinc supplementation for sleep quality, more clinical trials with larger sample sizes are needed to further explore the safety and efficacy of this intervention.

## AUTHOR CONTRIBUTIONS

**Mostafa Shahraki Jazinaki**: Methodology. **Alireza Gheflati**: Investigation; supervision. **Mohammad Reza Shadmand Foumani Moghadam**: Writing—original draft. **Saeid Hadi**: Writing—original draft. **Maryam Razavidarmian**: Data curation. **Masoud Yaghob Nezhad**: Investigation. **Hale Akhtari**: Investigation. **Mona Nematizadeh**: Investigation. **Mohammad Safarian**: Supervision.

## CONFLICT OF INTEREST STATEMENT

The authors declare no conflict of interest.

## ETHICS STATEMENT

The ethics committee of Mashhad University of Medical Sciences approved this review. All authors have read and approved the final version of the manuscript. The corresponding author had full access to all of the data in this study and takes complete responsibility for the integrity of the data and the accuracy of the data analysis. Also, the protocol for conducting this review is registered in the PROSPERO database with registration code CRD42023440955.

## TRANSPARENCY STATEMENT

The lead author Mohammad Safarian affirms that this manuscript is an honest, accurate, and transparent account of the study being reported; that no important aspects of the study have been omitted; and that any discrepancies from the study as planned (and, if relevant, registered) have been explained.

## Data Availability

The data that support the findings of this study are available on request from the corresponding author.
